# An Entity Extraction Pipeline for Medical Text Records Using Large Language Models: Analytical Study

**DOI:** 10.2196/54580

**Published:** 2024-03-29

**Authors:** Lei Wang, Yinyao Ma, Wenshuai Bi, Hanlin Lv, Yuxiang Li

**Affiliations:** 1 BGI Research Wuhan China; 2 Guangdong Bigdata Engineering Technology Research Center for Life Sciences BGI Research Shenzhen China; 3 Department of Obstetrics People’s Hospital of Guangxi Zhuang Autonomous Region Nanning China

**Keywords:** clinical data extraction, large language models, feature hallucination, modular approach, unstructured data processing

## Abstract

**Background:**

The study of disease progression relies on clinical data, including text data, and extracting valuable features from text data has been a research hot spot. With the rise of large language models (LLMs), semantic-based extraction pipelines are gaining acceptance in clinical research. However, the security and feature hallucination issues of LLMs require further attention.

**Objective:**

This study aimed to introduce a novel modular LLM pipeline, which could semantically extract features from textual patient admission records.

**Methods:**

The pipeline was designed to process a systematic succession of concept extraction, aggregation, question generation, corpus extraction, and question-and-answer scale extraction, which was tested via 2 low-parameter LLMs: Qwen-14B-Chat (QWEN) and Baichuan2-13B-Chat (BAICHUAN). A data set of 25,709 pregnancy cases from the People’s Hospital of Guangxi Zhuang Autonomous Region, China, was used for evaluation with the help of a local expert’s annotation. The pipeline was evaluated with the metrics of accuracy and precision, null ratio, and time consumption. Additionally, we evaluated its performance via a quantified version of Qwen-14B-Chat on a consumer-grade GPU.

**Results:**

The pipeline demonstrates a high level of precision in feature extraction, as evidenced by the accuracy and precision results of Qwen-14B-Chat (95.52% and 92.93%, respectively) and Baichuan2-13B-Chat (95.86% and 90.08%, respectively). Furthermore, the pipeline exhibited low null ratios and variable time consumption. The INT4-quantified version of QWEN delivered an enhanced performance with 97.28% accuracy and a 0% null ratio.

**Conclusions:**

The pipeline exhibited consistent performance across different LLMs and efficiently extracted clinical features from textual data. It also showed reliable performance on consumer-grade hardware. This approach offers a viable and effective solution for mining clinical research data from textual records.

## Introduction

Clinical text data have been widely recognized in data research due to their inclusion of multisource information [1,2] (eg, patient subjective statements, past objective facts, doctors’ diagnostic processes, and summary records). Extracting useful information from text data could serve as a crucial supplement to the study of disease progression; it could complement objective indicators dependent on laboratory tests and examinations [3], which has consistently been a hot research topic [4,5].

Historically, methods for text data extraction mainly include the following:

- Manual annotation: scales are designed based on clinical and research experience, followed by manual field extraction [6-8].
- Rule extraction: concepts from established knowledge base, such as *International Classification of Diseases, Tenth Revision* [9], are used for concept term extraction. This process is typically based on similarity algorithms and manual assistance to extract terms and their attributes (eg, negations and dependency relationships) [10].
- Named entity recognition or natural language processing algorithms: supervised learning methods, such as pretrained models like T5 [11], Bidirectional Encoder Representations from Transformers (BERT) [12], and BERT’s variants [13-15], with manual annotation to enhance semantic comprehension capabilities [16,17].

The task of extracting features from vast unstructured text presents itself as a daunting, labor-intensive, and time-consuming endeavor [18], for the following reasons:

- It is challenging to determine the dimension of extracted features initially, and from another perspective, confining the feature dimension means constraining the research scope from the outset [19].
- Given the inherent subjectivity and potential biases of recording subjects, solely relying on algorithms without annotation typically results in low accuracy and recall [20].
- Achieving higher accuracy with a broader feature scope, and the required human effort involved, is typically nonlinear [4], and the difficulty becomes apparent when confronted with massive real-world data.

The advent of large language models (LLMs) has paved a new path for the dilemma in clinical text extraction [21-23]. In the realm of natural language understanding research, generative large models, represented notably by ChatGPT [24] since 2022, have achieved unimaginable capabilities in semantic dimensions, leveraging the emergent intelligence from vast parameter scales. However, there are numerous considerations and limitations in their application, as follows:

- High-performing LLMs, such as OpenAI ChatGPT and Google Bard [25,26], are currently not open source, and patient data need to be submitted to their platform for analysis, presenting security challenges [27,28].
- Open-source LLMs with high intelligence generally require a large number of parameters (10-100 billion), which are hard to support on consumer-level graphics processing units (GPUs) [29].
- Low-parameter (around 10 billion) LLMs, typically require multistrategy support when dealing with tasks in certain vertical segments [30] (eg, fine-tuning, knowledge base or knowledge graph support, complex Chain of Thought (CoT) [31] along with its derivatives, and even global training) and are accompanied by various anomaly issues, including feature hallucination.

Although the application of LLM faces various potential limitations and challenges, as mentioned above, the foundational entity extraction and understanding capabilities of LLMs can still be used for low-cost extraction of clinical text data through meticulous prompt design, guidance combining CoT, and standardized examples [30,32].

In this study, we aimed to extract valuable features from a series of given patient admission records, which include the chief complaint and the medical histories. In light of this task, we introduced a modular LLM approach, which divides the entire extraction path into several smaller steps, with each modular LLM handling these basic steps automatically. We adopted the core idea of LLM agents [30] and self-consistency with CoT [33].

To experiment with this approach, we implemented 2 low-parameter LLMs in a local environment and compared their performances within a retrospective cohort of pregnancy to provide a reference that future researchers might draw upon.

## Methods

### Study Preparation

#### Data Sources

In this study, the text corpus was compiled from two primary sources:

1. Chief complaints and medical histories, exemplified in Multimedia Appendix 1, were extracted from inpatient admission records of an established cohort at the People’s Hospital of Guangxi Zhuang Autonomous Region in China. The established cohort for the preeclampsia risk study consisted of 25,709 pregnancies that received prenatal care between the 11th and 13th weeks of gestation from April 2012 to September 2021.
2. Clinical practice guidelines consisted of the 2018 guidelines from the American College of Obstetricians and Gynecologists [34] and the 2019 guidelines from the National Institute for Health and Care Excellence [35].

To ensure linguistic consistency, the entire corpus was maintained in Chinese.

#### Model Deployment

We deployed 2 most exemplary LLMs in the Chinese domain until September 2023 in an intranet security environment independently: Qwen-14B-Chat (QWEN) [36] and Baichuan2-13B-Chat (BAICHUAN) [37]. In the environment, the server cluster used NVIDIA DGX-A100 (2×40 G) GPU nodes. The QWEN used 29 GB of storage and 27 GB of GPU memory, while the BAICHUAN used 26 GB of storage and 28.9 GB of GPU memory. Both models operated solely on physically isolated GPUs, and access was facilitated through the OpenAI [38] format and FastChat [39]. The LLMs were built upon PyTorch 2.0, with the temperature set to 0 and max_token adjusted task by task.

#### Experimental Path

**Figure 1:**
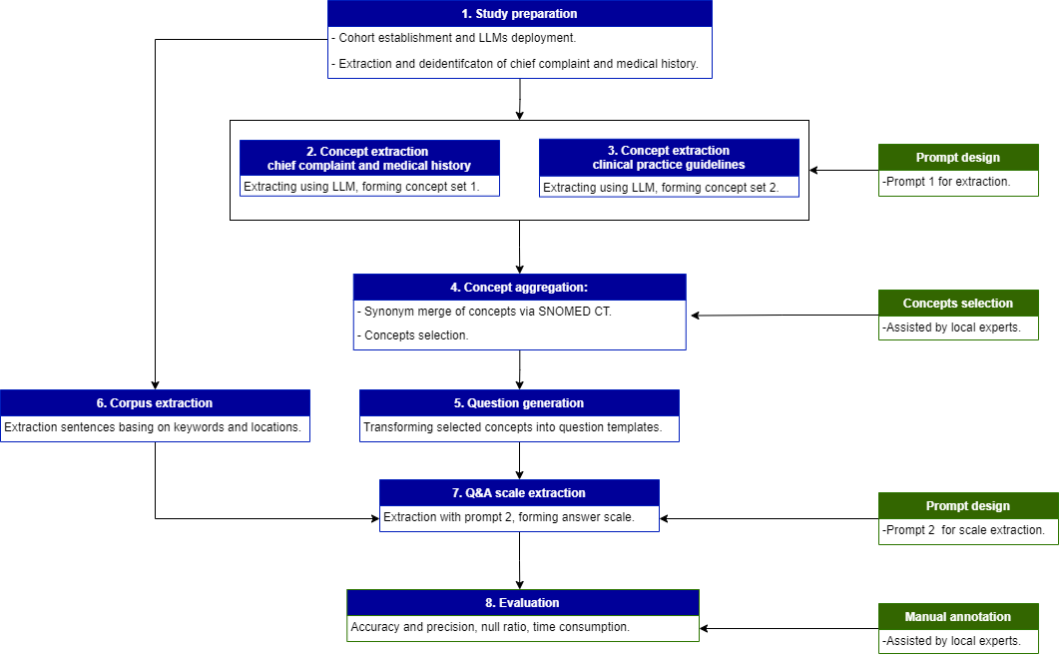
The flowchart of extraction approach. LLM: large language model; Q&A: question and answer; SNOMED CT: Systematised Nomenclature of Medicine Clinical Term.

In this study, we have introduced an approach that autonomously extracts valuable textual features. Diverging from traditional LLM applications, we used an “external-COT” strategy, dividing the process into several controllable steps, as illustrated in Figure 1.

The extraction approach could be divided into four parts: (1) concept preparation, that is, extracting existing concepts from the corpus and selecting concerning concepts; (2) corpus preparation, that is, deidentifying raw data and preparing the corpus in accordance with the selected concept; (3) prompt design for different LLM tasks; (4) question-and-answer (Q&A) scales, that is, transforming concepts into question templates and extracting corresponding scales by LLMs.

#### Prompt Design

The design of prompt templates is fundamental to efficient and accurate extraction. Prior to processing the entire data set, an initial evaluation was conducted on 100 observations to assess the effectiveness of the templates, allowing for continuous refinement of prompt strategies and orientations. An appropriate template was defined based on the following criteria: (1) absence of redundant content generation, (2) consistent and uniform efficiency, and (3) infrequent occurrence of feature hallucination.

We adopted a 4-paragraph structure, referring to the prompt engineering suggestions of QWEN and BAICHUAN, as follows:

1. Context section: defines the role and task, provides a basic understanding, and establishes a behavioral baseline for the model.
2. Instruction section: outlines the execution steps, uses the CoT methodology, and provides examples to ensure guided model operation.
3. Input data section: manages various inputs to meet diverse information needs.
4. Output indicator section: specifies the output format and standards, setting clear expectations for the output.

To avoid input bias, the prompt templates for QWEN and BAICHUAN were maintained without any modifications. In addition, we conducted experiments using 100 observations at different levels of concurrency to select the most optimal configuration.

#### Concept Extraction and Aggregation

We initially extracted all discernible concepts from chief complaints and medical histories using LLMs with a designed prompt 1, and concepts were retained only with a manifestation frequency exceeding 5% occurrences. To reduce potential attention bias and expand the range of identified concepts, we also included concepts from clinical practice guidelines related to preeclampsia, particularly the American College of Obstetricians and Gynecologists 2018 guidelines and the National Institute for Health and Care Excellence 2019 guidelines.

As we defined in prompt 1, the extracted concepts were formatted using the Systematised Nomenclature of Medicine Clinical Terms (SNOMED CT) vocabulary within the Clinical Findings and Observations domain [40].

To mitigate potential output errors from LLMs, such as concepts not belonging to the Clinical Findings and Observations domain, or even errors outside of the SNOMED CT vocabulary, we implemented a rule-based matching approach to filter extraction inaccuracies.

Furthermore, in this research, we aimed to extract concepts with diverse semantic expressions (including diagnoses, various medical histories, symptoms, observations, interventions, and types of examination). To accomplish this, local experts manually filtered out concepts embedded in structured text, such as dates or numbers.

#### Question Generation

After the extraction and aggregation of concepts, they were transformed into specific questions by LLMs as question templates for subsequent data extraction. In this section, we leveraged ChatGPT4.0 as a question generator to produce a basic set of questions, which were then refined by local experts for specificity based on its performance across 100 observations.

#### Q&A Scale Extraction

To avoid contextual and temporal event confusion leading to incorrect responses (eg, confusing current medical history with a past medical history or confusing the patient’s medical history with that of family members), we preextracted the corpus using two strategies: (1) based on the position of the question templates and (2) based on the sentence containing the concepts. The extracted corpus was then labeled with the corresponding question templates for the subsequent extraction of Q&A scales.

The refined corpus, combined with corresponding question templates, guided a systematic extraction process with 2 LLMs, forming Q&A scales for further application.

Each question probed the LLMs, and the extracted sentences formed the basis of the generated responses. This approach enabled a logical mapping of questions to relevant text, ultimately improving the accuracy and efficiency of feature extraction.

#### Evaluation

Given the practical constraints and the objective of minimizing manual intervention, it was unfeasible to validate all answer scales individually across a Q&A space containing 68 questions and 25,709 observations. Therefore, a 3-fold assessment strategy was developed, as explained in the sections that follow.

#### Accuracy and Precision

A subset of 1500 observations chosen at random was manually annotated in collaboration with local experts, serving as the gold standard. The precision of positive identifications by both LLMs was assessed against a specified benchmark.

#### Null Ratio

The null ratio of both LLMs was independently measured across all 25,709 observations. Empty or meaningless outputs (symbols and gibberish) were identified as null outputs, and the null ratio was then calculated as the proportion of such responses to the total.

#### Time Consumption

The efficiency of the extraction process was evaluated by measuring the time taken by the 2 LLMs to respond to the questions across all 25,709 observations.

### Ethical Considerations

The study was approved by the People’s Hospital of the Guangxi Zhuang Autonomous Region in China (KT-KJT-2021-67). The requirement for informed consent was waived, due to the retrospective nature of the study, and all clinical data were deidentified and anonymized.

## Results

### Path Decomposition Overview

#### Prompt Template

**Figure 2:**
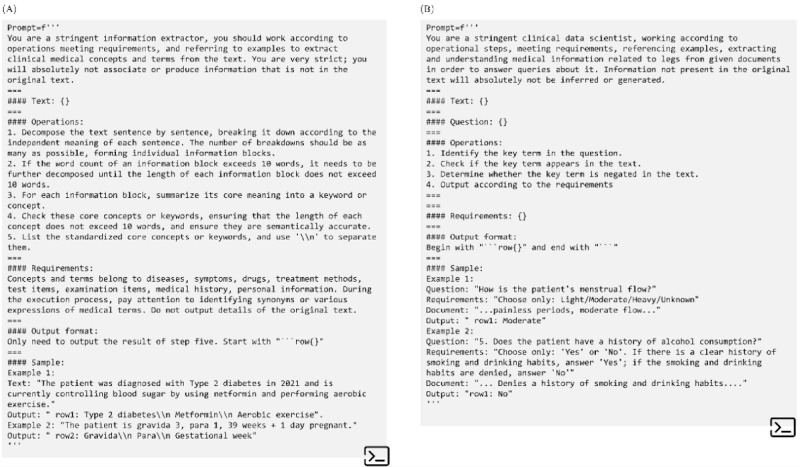
Prompt templates of information extraction and question-and-answer scales. A 4-paragraph structure was implemented for a prompt design using a few-shot Chain-of-Thought prompting. The original version, written in Chinese, was translated into English. (A) Extracting concepts and terms; (B) scaling questions. As both deployed large language models were pretrained with the *International Classification of Diseases, Tenth Revision* corpus, they could directly engage in concept extraction.

Through trials with the prompt template on 100 observations, we selected the template that demonstrated optimal consistency, as shown in Figure 2.

#### Merged Concepts

After merging all concepts, we filtered out those that appeared less than 5% of the time. A total of 117 concepts and terms were listed in Table S1 in Multimedia Appendix 2.

#### Question Template

Then we selected and transferred 68 concepts into question formats, for further Q&A scales. The detailed questions and their corresponding concepts are listed in Table S2 in Multimedia Appendix 2.

#### Scale Extraction

We identified that the optimal performance in Q&A scale extraction occurs with a concurrency of 3 requests, enhancing speed by 17.9% compared to a single request. Furthermore, we used a max_token restriction strategy, capping it at 20, to optimize inference speed.

Ultimately, within the 2D Q&A space formed by the answer scales, there were a total of 68 question columns and 25,709 observations (listed in Table 1).

We used accuracy and precision metrics for assessing the accuracy of LLMs across 1500 observations. Furthermore, we used 2 parameters—null ratio and time consumption—in 25,709 observations to evaluate the consistency and efficiency of the 2 LLMs, respectively.

**Table 1:** Question-and-answer scales for Qwen-14B-Chat (QWEN) and Baichuan2-13B-Chat (BAICHUAN).

Concepts	QWEN			BAICHUAN		
	Positive ratio (%)	Negative ratio (%)	Null ratio (%)	Positive ratio (%)	Negative ratio (%)	Null ratio (%)
Menstrual color	—^a^	—	0.00	—	—	0.67
Menstrual flow	—	—	0.00	—	—	0.80
Pregnancy weight gain^b^	—	—	1.60	—	—	2.53
Abdominal bloating	26.27	73.73	0.00	38.40	61.60	0.00
Abdominal pain	49.00	51.00	0.00	43.13	52.00	4.87
Amniocentesis	1.00	99.00	0.00	0.73	98.87	0.40
Aspirin use	1.40	98.60	0.00	1.40	98.60	0.00
Bilateral adnexal masses	4.87	95.13	0.00	0.73	99.20	0.07
Bilateral lower limb edema	51.53	48.47	0.00	4.87	95.13	0.00
Blood glucose screening	17.87	82.13	0.00	28.13	71.40	0.47
Cervical secretions	3.53	96.47	0.00	3.67	96.33	0.00
Chest tightness	2.87	97.13	0.00	4.40	95.53	0.07
Cold or flu	1.87	98.13	0.00	3.07	96.93	0.00
Convulsions	2.27	97.73	0.00	0.33	99.67	0.00
Dizziness	7.13	92.87	0.00	1.87	98.13	0.00
Drinking	0.00	100.00	0.00	0.00	100.00	0.00
Early pregnancy reaction or symptoms	85.93	14.07	0.00	47.53	52.13	0.33
Family history (asthma)	0.07	99.93	0.00	1.93	98.07	0.00
Family history (autoimmune disease)	0.20	99.80	0.00	3.07	96.93	0.00
Family history (diabetes mellitus)	1.67	98.33	0.00	2.33	97.67	0.00
Family history (drug allergy)	0.01	99.99	0.00	5.67	94.33	0.00
Family history (heart disease)	1.60	98.40	0.00	2.27	97.73	0.00
Family history (hematologic disease)	0.13	99.87	0.00	0.73	98.87	0.40
Family history (hypertension)	3.87	96.13	0.00	3.93	96.07	0.00
Family history (kidney disease)	0.07	99.93	0.00	1.40	98.53	0.07
Family history (mental illness)	0.07	99.93	0.00	0.47	99.47	0.07
Family history (neurological disease)	0.47	99.53	0.00	0.73	98.80	0.47
Family history (preeclampsia)	0.12	99.88	0.00	2.60	97.40	0.00
Family history (rheumatic disease)	0.07	99.93	0.00	1.47	98.53	0.00
Fetal paternal drinking history)	0.00	100.00	0.00	0.07	99.93	0.00
Fetal paternal history of genetic diseases	1.00	99.00	0.00	0.87	99.07	0.07
Fetal paternal smoking history	0.00	100.00	0.00	0.07	99.93	0.00
Fever	9.67	90.33	0.00	1.93	98.07	0.00
G6PD^c^	3.33	96.67	0.00	2.53	97.47	0.00
Headache	2.80	97.20	0.00	0.80	99.13	0.07
Insomnia	0.60	99.40	0.00	1.20	98.47	0.33
Mediterranean anemia screening	8.27	91.73	0.00	17.60	82.27	0.13
Palpitations	1.53	98.47	0.00	3.07	96.93	0.00
Personal history (antiphospholipid syndrome)	0.07	99.93	0.00	0.07	99.93	0.00
Personal history (chronic kidney disease)	0.80	99.20	0.00	1.07	98.93	0.00
Personal history (diabetes mellitus)	0.60	99.40	0.00	0.13	99.87	0.00
Personal history (drug allergy)	10.53	89.47	0.00	39.80	60.20	0.00
Personal history (dysmenorrhea)	24.40	75.60	0.00	21.20	78.80	0.00
Personal history (food allergy)	5.13	94.87	0.00	8.07	91.93	0.00
Personal history (heart disease)	1.67	98.33	0.00	0.47	99.53	0.00
Personal history (hematologic disease)	0.00	100.00	0.00	0.07	99.93	0.00
Personal history (hypertension)	7.40	92.60	0.00	0.93	99.07	0.00
Personal history (infectious disease)	1.93	98.07	0.00	3.80	96.20	0.00
Personal history (preeclampsia)	0.93	99.07	0.00	0.87	99.13	0.00
Personal history (surgery history)	35.67	64.33	0.00	36.27	63.67	0.07
Personal history (systemic lupus erythematosus)	0.20	99.80	0.00	0.20	99.80	0.00
Personal history (thalassemia)	1.00	99.00	0.00	0.80	99.13	0.07
Personal history (trauma history)	8.80	91.20	0.00	2.87	97.13	0.00
Personal history (viral hepatitis)	6.07	93.93	0.00	6.20	93.80	0.00
Poor pregnancy history (induced abortion)	0.07	99.93	0.00	0.13	99.87	0.00
Poor pregnancy history (miscarriage)	0.47	99.53	0.00	0.47	99.53	0.00
Poor pregnancy history (premature birth)	0.27	99.73	0.00	0.27	99.73	0.00
Prenatal screening	31.13	68.87	0.00	15.87	83.40	0.73
Regular prenatal check-ups	96.20	3.80	0.00	96.80	3.13	0.07
Smoking	0.07	99.93	0.00	0.07	99.93	0.00
Threatened abortion	6.20	93.80	0.00	5.80	94.00	0.20
Umbilical cord blood ratio	0.00	100.00	0.00	0.00	100.00	0.00
Use of antihypertensive drugs	1.73	98.27	0.00	2.13	97.87	0.00
Use of progestogen drugs	13.47	86.53	0.00	14.40	85.53	0.07
Vaginal bleeding	81.07	18.93	0.00	22.60	77.27	0.13
Vaginal discharge	33.00	67.00	0.00	48.47	51.53	0.00
Vaginal infection	25.27	74.73	0.00	16.20	82.73	1.07
Vaginal secretions	16.60	83.40	0.00	16.73	83.27	0.00

^a^Not applicable.

^b^The mean pregnancy weight gain was 13.73 (SD 24.12) for QWEN and 13.75 (SD 31.28) for BAICHUAN.

^c^G6PD: glucose-6-phosphate dehydrogenase.

### Evaluation Metrics

#### Accuracy and Precision

**Figure 3:**
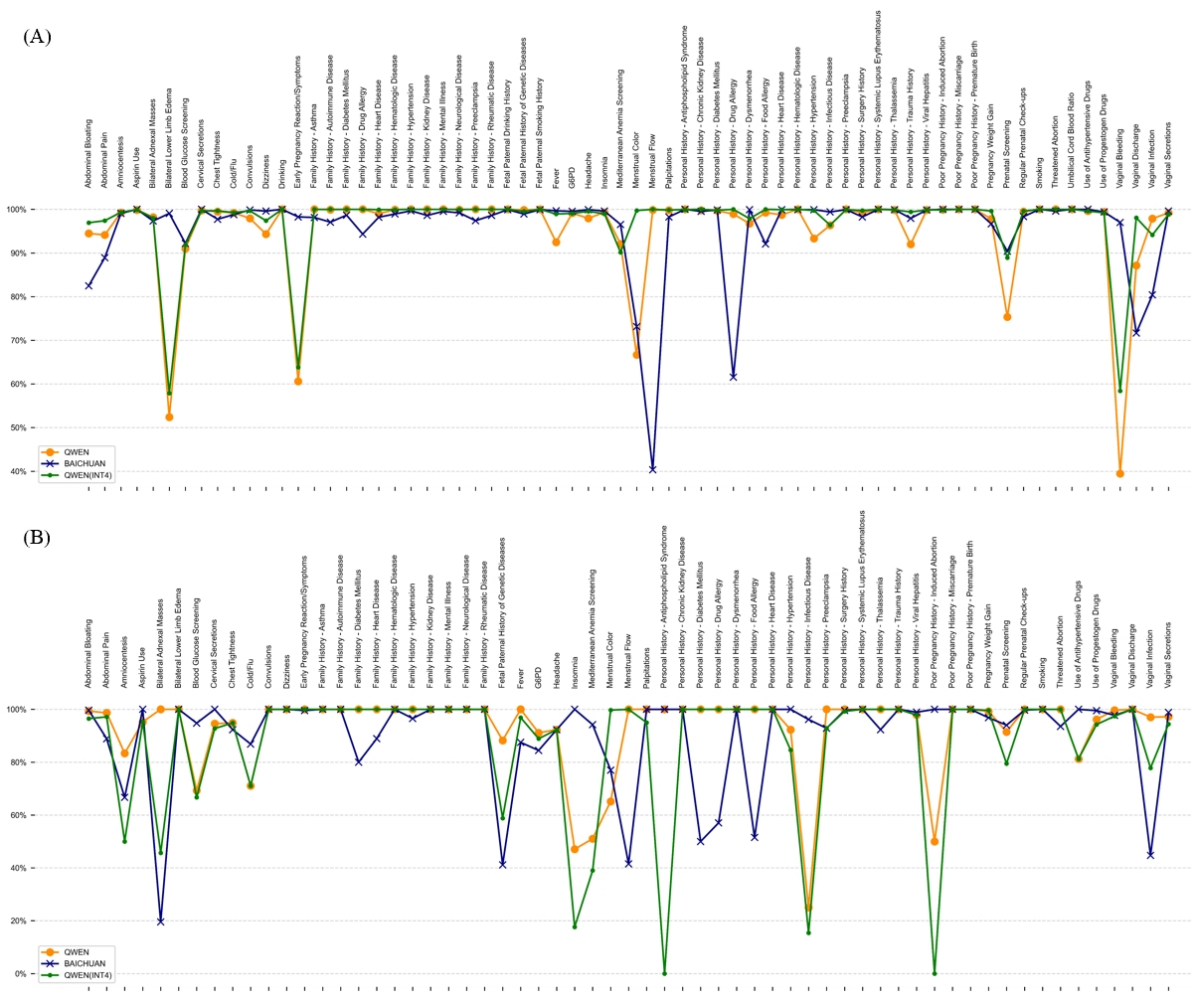
Accuracy and precision in the question-and-answer space. With the local expert’s annotation of 1500 observations, parts A and B showcase a comparison of the accuracy and precision of QWEN, BAICHUAN, and QWEN(INT4) across various concepts. Our findings reveal that the performance trends of large language models are nearly uniform across different concepts in terms of accuracy while showing a discernible variation in precision. G6PD: glucose-6-phosphate dehydrogenase.

Figure 3A and 3B and Figure S1 (parts A and B) in Multimedia Appendix 2 illustrate the Q&A space for a sample chunk extracted by QWEN and BAICHUAN with the comparison of manual annotation. The figures demonstrate the exceptional accuracy and precision of QWEN and BAICHUAN. QWEN attained an average accuracy of 95.52% and an average precision of 92.93%, whereas BAICHUAN displayed an average accuracy of 95.86% and an average precision of 90.08%. These figures clearly indicate that the 2 LLMs have more concentrated errors in specific concepts, and overall, they achieve high levels of precision in most extractions.

LLMs demonstrated consistent performance across most questions and excelled in binary, well-defined medical history questions, often reaching 100% accuracy and precision. However, the accuracy performance varied significantly when dealing with questions that involved semantic ambiguities or definitional uncertainties. This inconsistency might be tied to the LLM’s training and inference alignment. Notable disparities were observed in questions pertaining to menstrual color (QWEN: 1000/1500, 66.7%; BAICHUAN: 1097/1500, 73.1%), early pregnancy symptoms (QWEN: 909/1500, 60.7%; BAICHUAN: 1474/1500, 98.3%), vaginal bleeding (QWEN: 593/1500, 39.5%; BAICHUAN: 1455/1500, 97%), bilateral lower limb edema (QWEN: 786/1500, 52.4%; BAICHUAN: 1486/1500, 99.7%), and menstrual flow (QWEN: 1498/1500, 99.8%; BAICHUAN: 605/1500, 40.3%).

Apart from the above, the precision inconsistency performance of concepts could be attributed to their low true positive rate, like insomnia (QWEN: 3/17, 17.7%; BAICHUAN: 8/17, 47.1%), personal history—antiphospholipid syndrome (QWEN: 0/2, 0%; BAICHUAN: 1/2, 50%), and poor pregnancy history—induced abortion (QWEN: 1/2, 50%; BAICHUAN: 2/2, 100%). The exact precision is listed in Table S3 in Multimedia Appendix 2.

#### Null Ratio

As depicted in Table 1, both LLMs demonstrated superior performance with minimal null ratios. Specifically, QWEN (Figure S1A in Multimedia Appendix 2) exhibited a mean null ratio of 0.02%, in contrast to BAICHUAN (Figure S1B in Multimedia Appendix 2), which recorded a slightly higher null ratio of 0.21%. Failure of QWEN extraction was only in pregnancy weight gain (411/25,709, 1.60%), but failures of BAICHUAN extraction were mainly in symptoms (abdominal pain: 1252/25,707, 4.87%; vaginal infection: 275/25,709, 1.07%).

#### Time Consumption

**Figure 4:**
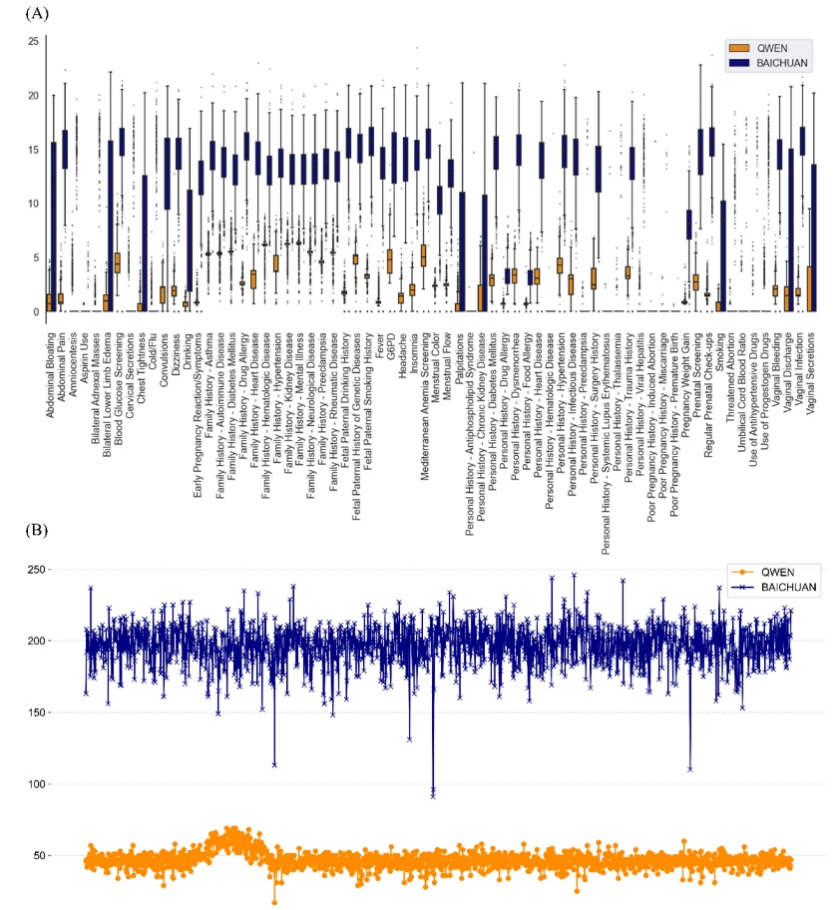
Time consumption of question-and-answer (Q&A) scales for QWEN and BAICHUAN, measured in seconds. (A) Comparative distribution of time consumption for QWEN and BAICHUAN per concept, and QWEN exhibited notably lower extraction times across various Q&A scales compared to BAICHUAN. (B) Comparative distribution of the time consumption for 2 large language models per observation. G6PD: glucose-6-phosphate dehydrogenase.

We conducted a comparative analysis of the time performance between QWEN and BAICHUAN on various Q&A scales, discovering that BAICHUAN consistently exhibits higher time consumption across almost all scales, reaching up to 4 times that of QWEN, as illustrated in Figure 4B.

Figure 4A compares the time consumption of LLMs in extracting diverse concepts. Although there were significant differences across different concepts, overall, the LLMs demonstrated a consistent performance across these concepts. For queries with clear definitions and concise corpora, such as drug usage and previous pregnancy history, the time consumed was minimal. In the category of medical history, both models exhibited uniform and stable performances (QWEN and BAICHUAN both revealed a time consumption ratio of 1:3).

### Additional Research

In clinical practice, to address scenarios of resource constraints, we used a quantified version of the LLM in our study to validate the applicability of this approach. We used an official-release INT4 version of QWEN, QWEN(INT4). The model was deployed on an NVIDIA RTX 3090 GPU (24 GB).

With the same approach listed above, the performance of QWEN(INT4) achieved even better performance. Figure 3 and Figure S1C in Multimedia Appendix 2 demonstrate that the average accuracy of QWEN(INT4) is 97.28%, accompanied by a null ratio of 0%.

Despite a notable correlation in performance extraction between QWEN(INT4) and QWEN, QWEN(INT4) demonstrated superior efficiency on limited hardware, with an average of 31 seconds per observation, compared to 47 seconds for QWEN and 312 seconds for BAICHUAN.

## Discussion

### Principal Findings

In this study, the extracted scales incorporated not only the conventional features of interest but also less frequently mentioned dimensions in previous cohorts or guidelines. These included food and drug allergies (6.6% for food allergy and 25.2% for drug allergy), certain pregnancy symptoms (average positive ratio of 0.9% for insomnia and 2.3% for palpitations), menstrual conditions (22.8% for dysmenorrhea), medical history (1% for asthma family history and 0.27% for mental illness family history), and gestational intervention (13.93% for progestogen and 1.4% for aspirin).

As a naturally recruited cohort of pregnancy, the extracted features show comparable proportions or trends compared with similar studies, such as systemic lupus erythematosus (average positive ratio of 0.20% vs 0.03%-0.23% of similar cohorts [41,42]) and antiphospholipid syndrome (average positive ratio of 0.08% vs 0.02%-0.12% of similar cohorts [43]), thereby corroborating the accuracy of our approach.

Additionally, certain scale deviations were revealed compared to similar studies, notably in fetal paternal smoking history (average positive ratio of 0.04% vs approximately 28.1%-40% in similar studies [44,45]). Although these deviations were few, we conducted a sample retracing to the original texts and determined that the extraction approach was not at fault and accurately reflected the original data. This discrepancy highlights persistent concerns [46] regarding the data quality in inpatient documentation, originating from patient self-reports and physician documentation, and vulnerability to recall and inquiry bias. Documentation varies among patients, influenced not only by patient conditions but also by physicians’ writing habits. Thus, we regard our approach as a preexperimental data analysis. Despite the presence of biases or missing dimensions, the approach uncovers several dimensions absent in structured medical texts, and valuable insights could still be extracted from the data with appropriate statistics [47]. In clinical practice, preliminary interviews with documenting physicians are recommended prior to the selection of concepts to enhance data quality and mitigate potential biases.

In the context of the extraction process, even when deployed solely on a standard consumer-grade GPU (NVIDIA RTX 3090), the QWEN(INT4) completed the extraction of 25,709 observations and 68 features within 15 calendar days, averaging 48.9 seconds per observation. In practical applications, deploying 2 instances of QWEN(INT4) on a single graphics card, coupled with an additional deployment in CPU [36], is hypothesized to reduce the extraction to approximately 7 days. Furthermore, multi-GPU server clusters, prevalent in clinical environments, could markedly reduce processing times, potentially to the scale of hours.

**Figure 5:**
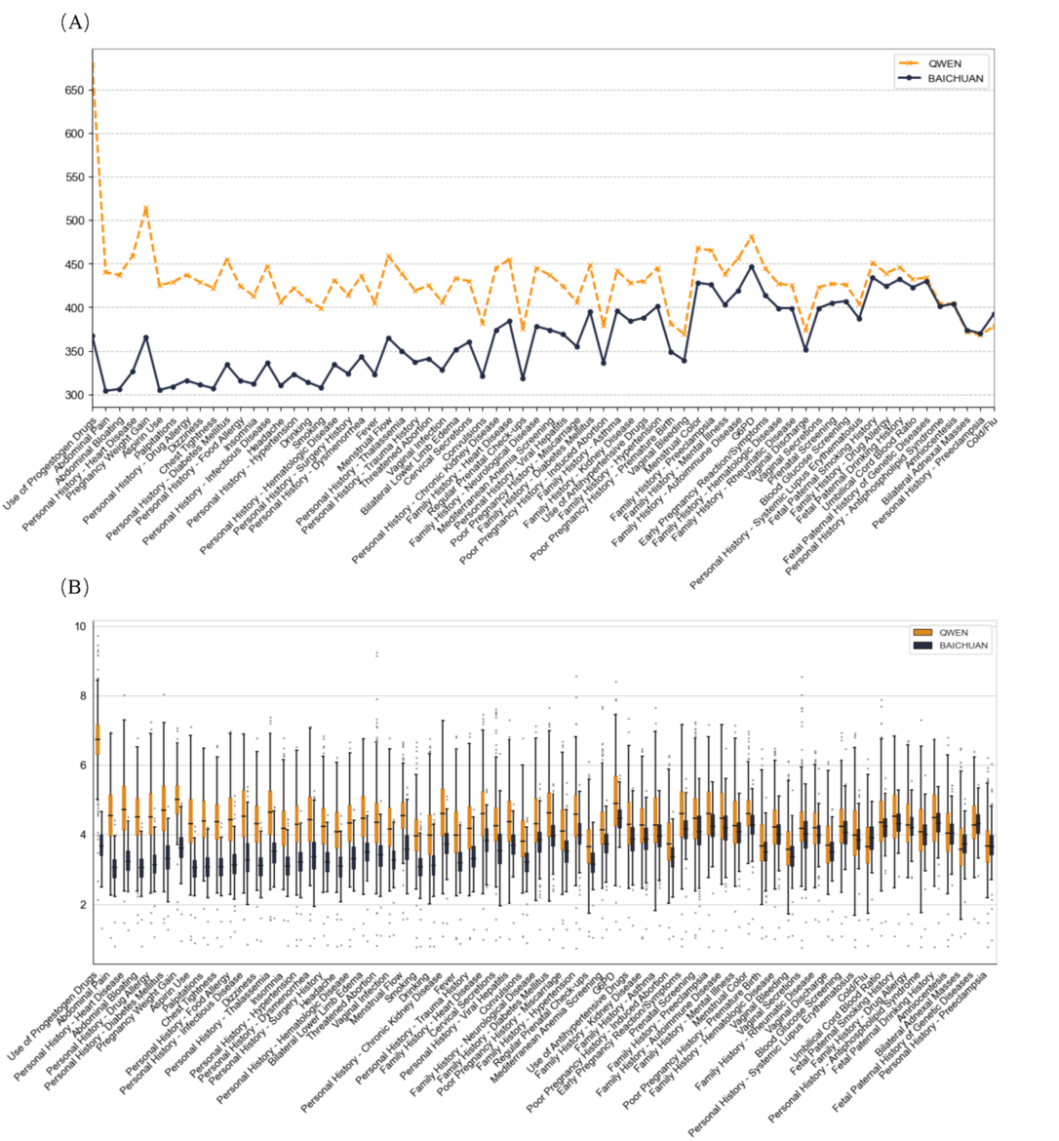
Approach performance when omitting corpus extraction step. (A) The average time consumed per question-and-answer (Q&A) interaction over 300 observations for both models. (B) Comparing the distribution of time consumption for QWEN and BAICHUAN in a single observation per Q&A scale. G6PD: glucose-6-phosphate dehydrogenase.

In our study, we experimented with omitting the corpus extraction step, directly using the long text of each observation’s chief complaints and medical histories as raw data for Q&A scale extraction. However, the experiment yielded poor performance in accuracy, precision, and time consumption, as illustrated in Figure 5 and Figure S2 in Multimedia Appendix 2.

These limitations appear significantly correlated with the current technological constraints of LLMs [32], which tend to generate “feature hallucinations” more frequently when processing extensive texts [48], leading to the loss of critical information. We believe that this issue will be resolved as the technology continues to evolve [49].

### Limitations

In our experimental validation, we selected a limited set of concepts, comprising only 68 items, to balance the consideration of time constraints. Despite our efforts to encompass a broad scope, some dimensions inevitably remain unaddressed, which is a limitation in verifying efficiency and accuracy across all dimensions.

Furthermore, the raw data in this study was sourced exclusively from a single hospital, spanning nearly a decade. This duration, while significant, introduces limitations in the generalizability of our approach.

Additionally, the approach used only 2 LLMs. Although we anticipate that future LLMs will be compatible with the current approach, this assumption necessitates further experimental validation.

### Conclusions

Our proposed approach offers a potential methodology for clinical text data analysis. It involves extracting and summarizing concepts from the comprehensive text of a defined population, thus selecting research directions of interest, and eventually generating analyzable features for the cohort. This approach demonstrates notable precision and could provide substantial data support for future research endeavors.

## Appendix

Multimedia Appendix 1 A sample of chief complaints and medical histories.

Multimedia Appendix 2 Additional statistics.

## Abbreviations

BERT: Bidirectional Encoder Representations from Transformers
CoT: Chain of Thought
GPU: graphics processing unit
LLM: large language model
Q&A: question and answer
SNOMED CT: Systematised Nomenclature of Medicine Clinical Term

## Data Availability

The data sets generated and analyzed during this study are not publicly available due to privacy or ethical restrictions but are available on request from the corresponding author.
