# Supplementary material for: An Entity Extraction Pipeline for Medical Text Records Using Large Language Models: Analytical Study
Source: J Med Internet Res. 2024 Mar 29;26:e54580. doi: 10.2196/54580 (PMC11015372; doi:10.2196/54580)
Supplement: Multimedia Appendix 1 [file jmir_v26i1e54580_app1.docx]

### A sample of chief complain and medical histories.

#### Translated version.

This material contains a synthesized example of a patient's admission record, translated from the original Chinese text, and is entirely fictionalized with no real patient data or identifiable private information included.

**Chief Complaint:**

Amenorrhea of 36 weeks and 5 days, vaginal fluid discharge for over 1 hour.

**Present Illness History:**

The patient complains of historically irregular menstruation, 6-7/37-45 days, with the last menstrual period on December 31, 2019. On March 18, 2020, a B-ultrasound of the uterus and adnexa in our hospital showed: intrauterine early pregnancy, visible fetal heartbeat, and a small cyst in the right adnexal area measuring about 15×10mm. According to B-ultrasound, the revised expected date of delivery is October 24, 2020. On March 27, 2020, a health check-up card was established at our hospital, with an initial diagnosis blood pressure of 98/70mmHg. The patient experienced significant early pregnancy symptoms like nausea and fatigue, which disappeared on their own after about 3 months of pregnancy. During the health check-up, tests for routine blood and urine, liver and kidney functions, electrolytes, coagulation function, eugenics eight items, hepatitis B two and a half pairs, immune four items, glucose-6-phosphate dehydrogenase, vaginal discharge routine, Ureaplasma urealyticum + gonococcus + Chlamydia trachomatis DNA determination showed no significant abnormalities. Free thyroid function three items: TSH 5.16 uIU/ml; Free T4 11.05 pmol/L; Free T3 5.48 pmol/L. The endocrinology outpatient clinic of our hospital diagnosed "pregnancy with hypothyroidism" and prescribed oral levothyroxine 1.25 tablets to treat hypothyroidism. Thyroid function was monitored during pregnancy and was acceptable. Both partners screened negative for thalassemia. On April 17, 2020, fetal NTB ultrasound showed: intrauterine pregnancy, single live fetus, early pregnancy, equivalent to 12 weeks and 6 days of gestation, CRL 65mm, NT 0.8mm. Mid-term Down syndrome screening all indicated low risk. From about 4 months of pregnancy, the patient began to feel fetal movements, which continue to the present. On June 30, 2020, a fetal four-dimensional color ultrasound at our hospital indicated: BPD 47mm, HC 183mm, a single live fetus in the uterus, mid-pregnancy, equivalent to 22 weeks of gestation; no significant complex malformations were observed in the fetal heart; fetal umbilical blood flow ratio showed no significant abnormalities. On July 20, 2020, a fetal B-ultrasound at our hospital indicated: BPD 56mm, HC 218mm, a single live fetus in the uterus, mid-pregnancy, breech position, according to various fetal biometric measurements, equivalent to 24 weeks and 5 days of gestation, biparietal diameter equivalent to 23 weeks and 1 day of gestation; no significant complex malformations were observed in the fetal heart; fetal umbilical blood flow ratio showed no significant abnormalities; placental function level 0; the fetal umbilical cord was wrapped around the neck twice. Because the biparietal diameter was less than 2 weeks of gestational age, umbilical blood puncture was performed at 26 weeks of pregnancy for chromosomal karyotype analysis, which showed no abnormalities. OGTT test was performed: 4.45-10.21-9.45mmol/l. Diagnosed with "gestational diabetes", dietary control was administered under the guidance of our hospital's nutrition department, and blood sugar control was acceptable. On August 20, 2020, a fetal system B-ultrasound at our hospital indicated: BPD 67mm, HC 275mm, AC 258mm, HL 51mm, FL 57mm, the placenta is located on the posterior wall of the uterus, the maximum anteroposterior diameter of the amniotic fluid is 47mm. A single live fetus in the uterus, late pregnancy, breech position, according to various fetal biometric measurements, equivalent to 29 weeks and 3 days of gestation; no significant complex malformations were observed in the fetal heart; fetal umbilical blood flow ratio showed no significant abnormalities; placental function level 0; the fetal umbilical cord was wrapped around the neck once. GBS test was performed in late pregnancy, indicating negative. Around 20:40 on October 1, 2020, a small amount of vaginal fluid was discharged, occasional abdominal distension was noted, no abdominal pain, no vaginal bleeding, self-perceived normal fetal movements, and the patient is now visiting our hospital. There have been no recent discomforts such as chills, fever, dry cough, sore throat, nasal congestion, runny nose, diarrhea, muscle aches, etc. The emergency department’s preliminary diagnosis is "1. Premature rupture of membranes 2. 36 weeks + 5 days of gestation (G1P0) 3. Vertex presentation 4. Gestational diabetes 5. Pregnancy with hypothyroidism 6. Umbilical cord entanglement" and the patient was admitted to the hospital. During pregnancy, the patient denies exposure to toxins and radiation, denies chills, fever, dizziness, blurred vision, denies abdominal distension, abdominal pain, denies chest tightness, palpitations, and other discomforts, is in good spirits, has a good appetite, sleeps well, has normal bowel movements, and gained 13kg during pregnancy.

**Past History:**

Previously healthy, denies a history of hypertension, diabetes, blood diseases, and heart disease. Denies history of hepatitis and other infectious diseases like tuberculosis, denies significant trauma history, denies history of blood transfusion, denies food and drug allergies, vaccination history is unclear, and review of other systems revealed no abnormalities.

**Personal History:**

Born and resides in her native place, denies long-term residence history in other places, denies bad habits like smoking and drinking.

**Menstrual and Obstetric History:**

Menarche at 13 years old, menstrual period of 6-7 days, menstrual cycle of 37-45 days, last menstrual period: December 31, 2019. The menstrual volume is medium, with dark red color, and no dysmenorrhea. Has a steady sexual partner, G1P0.

**Family History:**

Parents are healthy, denies family history of genetic diseases and infectious diseases.

#### Original Chinese version.

主诉： 停经36周5天，阴道流液超过1小时。

现病史： 患者表示其月经史不规则，周期为6-7/37-45天，末次月经时间为2019年12月31日。2020年3月18日，在我院进行子宫和附件的B超检查，结果显示：子宫内早孕，可见胎心跳，右侧附件区有一个小囊肿，大小约为15×10mm。根据B超，修正预产期为2020年10月24日。2020年3月27日，患者在我院建卡，初诊血压98/70mmHg。患者在怀孕初期早孕反应强烈，如恶心和疲劳，孕约3个月后自然消失。在健康检查中，常规血液和尿液、肝肾功能、电解质、凝血功能、优生八项、乙肝两半对、免疫四项、葡萄糖-6-磷酸脱氢酶、阴道分泌物常规、尿素化脲酶杆菌+淋球菌+沙眼衣原体DNA检测均未见明显异常。自由甲状腺功能三项：TSH 5.16 uIU/ml；游离T4 11.05 pmol/L；游离T3 5.48 pmol/L。我院内门诊诊断为"妊娠伴甲减"，口服左旋甲状腺素1.25片以治疗甲减。在妊娠期间监测甲状腺功能，结果可接受。双方夫妻地中海贫血筛查均为阴性。2020年4月17日，胎儿NTB超声显示：子宫内妊娠，单胎活胎，早孕，相当于孕12周+6，CRL 65mm，NT 0.8mm。中期唐氏综合症筛查均表明低风险。从怀孕约4个月开始，患者开始感觉到胎动，至今仍然持续。2020年6月30日，我院进行的胎儿四维彩超显示：BPD 47mm，HC 183mm，子宫内单胎活胎，中孕，相当于孕22周；在胎儿心脏未见显著复杂畸形；胎儿脐血流比无显著异常。2020年7月20日，我院进行的胎儿B超显示：BPD 56mm，HC 218mm，子宫内单胎活胎，中孕，臀位，根据各项胎儿测量，相当于孕24周5天，顶骨径相当于孕23周1天；在胎儿心脏未见显著复杂畸形；胎儿脐血流比无显著异常；胎盘功能级别0；胎儿脐带绕颈两圈。由于顶骨径小于2周的孕期，在孕26周行脐血穿刺进行染色体分析，结果未见异常。进行了OGTT测试：4.45-10.21-9.45mmol/l。诊断为"妊娠糖尿病"，在我院营养科的指导下进行了饮食控制，血糖控制可接受。2020年8月20日，我院行胎儿B超：BPD 67mm，HC 275mm，AC 258mm，HL 51mm，FL 57mm，胎盘位于子宫后壁，羊水最大前后径为47mm。子宫内单胎活胎，晚孕，臀位，根据各项胎儿测量，相当于怀孕29周3天；在胎儿心脏未见显著复杂畸形；胎儿脐血流比无显著异常；胎盘功能级别0；胎儿脐带绕颈一圈。行晚孕GSB测试，结果为阴性。2020年10月1日，20:40左右，阴道排出少量液体，偶腹胀，无腹痛，无阴道出血，自觉胎动正常。近无寒冷、发热、干咳、喉咙痛、鼻塞、流鼻涕、腹泻、肌肉疼痛等不适。急诊科的初步诊断是"1.胎膜早破 2.36周+5天妊娠(孕1产0) 3.头位 4.妊娠糖尿病 5.妊娠伴甲减 6.脐带纠结"，患者已住院。在怀孕期间，患者否认有暴露于毒物和辐射的情况，否认有寒冷、发热、头晕、视觉模糊，否认有腹胀、腹痛，否认有胸闷、心悸等其他不适，精神状态良好，食欲好，睡眠正常，排便正常，怀孕期间体重增加13公斤。

既往病史： 既往健康状况良好，否认高血压、糖尿病、血液病和心脏病史。否认肝炎和其他传染病史，否认有重大创伤史，否认有输血史，否认食物和药物过敏，疫苗接种史不清，其他系统回顾未见异常。

个人史：出生并居住在本地，否认在其他地方的长期居住史，否认不良嗜好如吸烟和饮酒史。

月经和产科史： 初潮13岁，月经期6-7天，月经周期37-45天，最后一次月经：2019年12月31日。月经量中等，颜色暗红，没有痛经的情况。有稳定的性伴侣，孕1产0。

家族史： 父母健康，否认遗传病和传染病的家族史。
