# Supplementary material for: An Entity Extraction Pipeline for Medical Text Records Using Large Language Models: Analytical Study
Source: J Med Internet Res. 2024 Mar 29;26:e54580. doi: 10.2196/54580 (PMC11015372; doi:10.2196/54580)

**Table S1.** Merged concept sets and classification.

| **Class** | **Concepts from chief complaints and medical histories** | **Concepts from clinical practice guidelines** |
| --- | --- | --- |
| Basic Information | Occupation, Age, Education (status), Marital Status, Date of admission to hospital, Date of last menstrual period, Estimated date of delivery, Pregnancy status findings, Finding of number of pregnancies, Number of fetuses in utero, Menstrual cycle finding, Menstrual blood loss finding, Color of menstrual blood | Occupation, Age, Ethnic Group, Marital Status, Gestational age, Finding of number of pregnancies, Number of fetuses in utero, History of tobacco use, History of alcohol consumption behavior |
| Condition/State Present | Nausea, Vomiting, Anorexia, Sense of fetal movement, Pyrexia, Feeling of chest pressure, Palpitations, Abdominal pain, Abdominal bloating and distension, Common cold, Vaginal discharge, Bleeding, Dizziness, Headache, Convulsion, Edema of both lower legs, Antenatal screening, Mass of both adnexa, Vaginal infection, Vertex presentation of fetus | Assisted reproductive technology, Threat of spontaneous abortion, Insomnia, Early pregnancy symptoms, |
| Indicators Requiring Specific Values | Cord around neck, Amniotic fluid index, Diameter of fetal biparietal, Thickness of placenta, Grading of placental function | Change in weight, Length of fetal femur |
| Pregnancy Indicator Results | Finding of amniotic fluid, Non-invasive prenatal screening, Finding of chromosomal analysis of amniotic fluid, Result of Ureaplasma urealyticum test, Ultrasonography (scan), Three-dimensional color doppler sonography, Screening for glucose tolerance, Screening for thalassemia, Glucose-6-phosphate dehydrogenase measurement, Triplicate immunization status, Quantitation of Hepatitis B test, Coagulation test, Eugenic screening, Thyroid function study, Finding of cervical secretions, Screening for Down's syndrome, Vaginal swab culture | Three-dimensional color doppler sonography, Finding of fetal umbilical blood Doppler |
| Pregnancy Treatment Interventions | Progesterone, Dydrogesterone, Folic acid, Azithromycin, Aspirin, Methyldopa, Labetalol | Aspirin, Labetalol |
| History or Present Illness Description | Dysmenorrhea, Previous pre-eclampsia, Diabetes mellitus, Blood disorder, Heart disease, Thalassemia, Viral hepatitis, Chronic kidney disease, Tuberculosis, Injury, History of surgical procedure, Sexually transmitted infectious disease, Genetic disease, History of infectious disease, Food allergy, Drug allergy | Hypertension, Previous preterm delivery, Systemic lupus erythematosus, Antiphospholipid syndrome, History of spontaneous abortion, History of deliberate termination of pregnancy |
| Family History | Family history of cardiac disorder, Family history of diabetes mellitus, Family history of hematological disease, Family history of liver disease, Family history of kidney disease, Family history of mental health disorder, Family history of neurological disorder, Family history of rheumatologic disorder, Family history of endocrine disease, Family history of autoimmune disorder, Family history of allergy, Family history of sudden death, Family history of obesity, Family history of asthma, Family history of fracture, Family history of surgical procedure, Family history of adverse reaction to drug | Family history of hypertension  Family history of preeclampsia |
| Health Status of the Fetus Father | Hereditary Disease, Present Illness History, Smoking History, Drinking History | Smoking History, Drinking History |

**Table S2**. Concept and question list

|  | Concept | Question |
| --- | --- | --- |
| 1 | Abdominal Bloating | Did the patient experience abdominal bloating during pregnancy? Provide a reason. |
| 2 | Abdominal Pain | Did the patient experience abdominal pain during pregnancy? Provide a reason. |
| 3 | Amniocentesis | Was the patient's amniocentesis result abnormal during pregnancy? Provide a reason. |
| 4 | Aspirin Use | Has the patient ever used aspirin? Provide a reason. |
| 5 | Bilateral Adnexal Masses | Did the patient have an adnexal mass found during pregnancy? Provide a reason. |
| 6 | Bilateral Lower Limb Edema | Did the patient have edema in the lower extremities during pregnancy? Provide a reason. |
| 7 | Blood Glucose Screening | Is the patient's blood glucose screening or OGTT test result abnormal? Provide a reason. |
| 8 | Cervical Secretions | Is the patient's cervical secretion test result abnormal? Provide a reason. |
| 9 | Chest Tightness | Did the patient experience chest tightness during pregnancy? Provide a reason. |
| 10 | Cold/Flu | Did the patient catch a cold during pregnancy? Provide a reason. |
| 11 | Convulsions | Did the patient have convulsions during pregnancy? Provide a reason. |
| 12 | Dizziness | Did the patient experience dizziness during pregnancy? Provide a reason. |
| 13 | Drinking | Does the patient report a history of alcohol consumption? Provide a reason. |
| 14 | Early Pregnancy Reaction/Symptoms | Did the patient experience nausea, vomiting, anorexia, or other early pregnancy reactions? Provide a reason. |
| 15 | Family History - Asthma | Is there a history of asthma in the patient's family? Provide a reason. |
| 16 | Family History - Autoimmune Disease | Is there a history of autoimmune diseases in the patient's family? Provide a reason. |
| 17 | Family History - Diabetes Mellitus | Is there a history of diabetes in the patient's family? Provide a reason. |
| 18 | Family History - Drug Allergy | Is there an allergy history in the patient's family? Provide a reason. |
| 19 | Family History - Heart Disease | Is there a history of heart disease in the patient's family? Provide a reason. |
| 20 | Family History - Hematologic Disease | Is there a history of blood disorders in the patient's family? Provide a reason. |
| 21 | Family History - Hypertension | Is there a history of hypertension in the patient's family? Provide a reason. |
| 22 | Family History - Kidney Disease | Is there a history of kidney disease in the patient's family? Provide a reason. |
| 23 | Family History - Mental Illness | Is there a history of mental illness in the patient's family? Provide a reason. |
| 24 | Family History - Neurological Disease | Is there a history of neurological disease in the patient's family? Provide a reason. |
| 25 | Family History - Preeclampsia | Has there been a history of pre-eclampsia in the patient's family? Provide a reason. |
| 26 | Family History - Rheumatic Disease | Is there a history of rheumatic disease in the patient's family? Provide a reason. |
| 27 | Fetal Paternal Drinking History | Does the physiological father of the fetus have a history or habit of drinking alcohol? Provide a reason. |
| 28 | Fetal Paternal History of Genetic Diseases | Does the physiological father of the fetus have a history of genetic diseases? Provide a reason. |
| 29 | Fetal Paternal Smoking History | Does the physiological father of the fetus have a history or habit of smoking? Provide a reason. |
| 30 | Fever | Did the patient have a fever during pregnancy? Provide a reason. |
| 31 | G6PD | Is the patient's glucose-6-phosphate dehydrogenase (G6PD) level abnormal? Provide a reason. |
| 32 | Headache | Did the patient experience headaches during pregnancy? Provide a reason. |
| 33 | Insomnia | Did the patient suffer from insomnia during pregnancy? Provide a reason. |
| 34 | Mediterranean Anemia Screening | Is the patient's thalassemia screening result abnormal? Provide a reason. |
| 35 | Menstrual Color | What is the color of the patient's menstruation? (Choose from: light red/red/dark red/other/unknown) |
| 36 | Menstrual Flow | What is the patient's menstrual volume? (Choose from: light/medium/heavy/unknown) |
| 37 | Palpitations | Did the patient experience palpitations during pregnancy? Provide a reason. |
| 38 | Personal History - Antiphospholipid Syndrome | Does the patient have a history of antiphospholipid syndrome? Provide a reason. |
| 39 | Personal History - Chronic Kidney Disease | Does the patient have a history of chronic kidney disease? Provide a reason. |
| 40 | Personal History - Diabetes Mellitus | Does the patient have a history of diabetes? Provide a reason. |
| 41 | Personal History - Drug Allergy | Does the patient admit to allergies to certain medications? Provide a reason. |
| 42 | Personal History - Dysmenorrhea | Does the patient experience dysmenorrhea? Provide a reason. |
| 43 | Personal History - Food Allergy | Does the patient admit to allergies to certain foods? Provide a reason. |
| 44 | Personal History - Heart Disease | Does the patient have a history of heart disease? Provide a reason. |
| 45 | Personal History - Hematologic Disease | Does the patient have a history of blood disorders? Provide a reason. |
| 46 | Personal History - Hypertension | Does the patient have a history of hypertension? Provide a reason. |
| 47 | Personal History - Infectious Disease | Does the patient admit to a history of infectious diseases (such as tuberculosis, gonorrhea, syphilis, etc.)? Provide a reason. |
| 48 | Personal History - Preeclampsia | Does the patient have a history of pre-eclampsia? Provide a reason. |
| 49 | Personal History - Surgery History | Does the patient have a history of surgery? Provide a reason. |
| 50 | Personal History - Systemic Lupus Erythematosus | Does the patient have a history of systemic lupus erythematosus? Provide a reason. |
| 51 | Personal History - Thalassemia | Does the patient have a history of thalassemia? Provide a reason. |
| 52 | Personal History - Trauma History | Does the patient have a history of trauma? Provide a reason. |
| 53 | Personal History - Viral Hepatitis | Does the patient have a history of viral hepatitis? Provide a reason. |
| 54 | Poor Pregnancy History - Induced Abortion | Does the patient admit to a history of induced abortion? Provide a reason. |
| 55 | Poor Pregnancy History - Miscarriage | Does the patient admit to a history of miscarriage? Provide a reason. |
| 56 | Poor Pregnancy History - Premature Birth | Does the patient admit to a history of preterm birth? Provide a reason. |
| 57 | Pregnancy Weight Gain | What is the patient's pregnancy weight change? (Answer with a number only.) |
| 58 | Prenatal Screening | Was the patient's Down syndrome screening or non-invasive DNA test result abnormal during pregnancy? Provide a reason. |
| 59 | Regular Prenatal Check-ups | Did the patient undergo regular prenatal check-ups during pregnancy? Provide a reason. |
| 60 | Smoking | Does the patient report a personal history of smoking? Provide a reason. |
| 61 | Threatened Abortion | Does the patient have a history of threatened abortion? Provide a reason. |
| 62 | Umbilical Cord Blood Ratio | Is the fetal umbilical cord blood ratio result of the patient abnormal? Provide a reason. |
| 63 | Use of Antihypertensive Drugs | Has the patient ever used antihypertensive medication? Provide a reason. |
| 64 | Use of Progestogen Drugs | Has the patient ever used progestogen medication? Provide a reason. |
| 65 | Vaginal Bleeding | Did the patient experience vaginal bleeding during pregnancy? Provide a reason. |
| 66 | Vaginal Discharge | Did the patient experience vaginal discharge during pregnancy? Provide a reason. |
| 67 | Vaginal Infection | Did the patient experience vaginal infections during pregnancy? Provide a reason. |
| 68 | Vaginal Secretions | Is the patient's vaginal secretion test result abnormal? Provide a reason. |

**Table S3**. LLMs Q&A scales precision.

| **Concept** | **QWEN** | **BAICHUAN** | **QWEN （INT4）** | **True positive** |
| --- | --- | --- | --- | --- |
| Abdominal Bloating | 313 | 314 | 304 | 315 |
| Abdominal Pain | 656 | 591 | 646 | 665 |
| Amniocentesis | 5 | 4 | 3 | 6 |
| Aspirin Use | 20 | 21 | 20 | 21 |
| Bilateral Adnexal Masses | 46 | 9 | 21 | 46 |
| Bilateral Lower Limb Edema | 59 | 59 | 59 | 59 |
| Blood Glucose Screening | 239 | 327 | 230 | 345 |
| Cervical Secretions | 52 | 55 | 51 | 55 |
| Chest Tightness | 37 | 36 | 37 | 39 |
| Cold/Flu | 27 | 33 | 27 | 38 |
| Convulsions | 3 | 3 | 3 | 3 |
| Dizziness | 22 | 22 | 22 | 22 |
| Early Pregnancy Reaction/Symptoms | 698 | 695 | 698 | 698 |
| Family History - Asthma | 1 | 1 | 1 | 1 |
| Family History - Autoimmune Disease | 2 | 2 | 2 | 2 |
| Family History - Diabetes Mellitus | 25 | 20 | 25 | 25 |
| Family History - Heart Disease | 9 | 8 | 9 | 9 |
| Family History - Hematologic Disease | 1 | 1 | 1 | 1 |
| Family History - Hypertension | 58 | 56 | 58 | 58 |
| Family History - Kidney Disease | 1 | 1 | 1 | 1 |
| Family History - Mental Illness | 1 | 1 | 1 | 1 |
| Family History - Neurological Disease | 6 | 6 | 6 | 6 |
| Family History - Rheumatic Disease | 1 | 1 | 1 | 1 |
| Fetal Paternal History of Genetic Diseases | 15 | 7 | 10 | 17 |
| Fever | 32 | 28 | 31 | 32 |
| G6PD | 41 | 38 | 40 | 45 |
| Headache | 12 | 12 | 12 | 13 |
| Insomnia | 8 | 17 | 3 | 17 |
| Mediterranean Anemia Screening | 123 | 227 | 94 | 241 |
| Menstrual Color | 920 | 1087 | 1408 | 1412 |
| Menstrual Flow | 1428 | 593 | 1428 | 1428 |
| Palpitations | 20 | 20 | 19 | 20 |
| Personal History - Antiphospholipid Syndrome | 1 | 1 | 0 | 1 |
| Personal History - Chronic Kidney Disease | 9 | 9 | 9 | 9 |
| Personal History - Diabetes Mellitus | 4 | 2 | 4 | 4 |
| Personal History - Drug Allergy | 142 | 81 | 142 | 142 |
| Personal History - Dysmenorrhea | 317 | 317 | 317 | 317 |
| Personal History - Food Allergy | 66 | 34 | 66 | 66 |
| Personal History - Heart Disease | 6 | 6 | 6 | 6 |
| Personal History - Hypertension | 12 | 13 | 11 | 13 |
| Personal History - Infectious Disease | 13 | 50 | 8 | 52 |
| Personal History - Preeclampsia | 14 | 13 | 13 | 14 |
| Personal History - Surgery History | 523 | 521 | 519 | 523 |
| Personal History - Systemic Lupus Erythematosus | 3 | 3 | 3 | 3 |
| Personal History - Thalassemia | 13 | 12 | 13 | 13 |
| Personal History - Trauma History | 12 | 12 | 12 | 12 |
| Personal History - Viral Hepatitis | 91 | 92 | 91 | 93 |
| Poor Pregnancy History - Induced Abortion | 1 | 2 | 0 | 2 |
| Poor Pregnancy History - Miscarriage | 7 | 7 | 7 | 7 |
| Poor Pregnancy History - Premature Birth | 4 | 4 | 4 | 4 |
| Pregnancy Weight Gain | 1441 | 1406 | 1446 | 1452 |
| Prenatal Screening | 107 | 110 | 93 | 117 |
| Regular Prenatal Check-ups | 1435 | 1431 | 1435 | 1435 |
| Smoking | 1 | 1 | 1 | 1 |
| Threatened Abortion | 93 | 87 | 93 | 93 |
| Use of Antihypertensive Drugs | 26 | 32 | 26 | 32 |
| Use of Progestogen Drugs | 201 | 208 | 197 | 209 |
| Vaginal Bleeding | 309 | 303 | 302 | 310 |
| Vaginal Discharge | 302 | 302 | 302 | 302 |
| Vaginal Infection | 358 | 165 | 287 | 369 |
| Vaginal Secretions | 244 | 248 | 237 | 251 |

Figure S1 Overview of comparation between LLMs outputs and expert’s annotation.


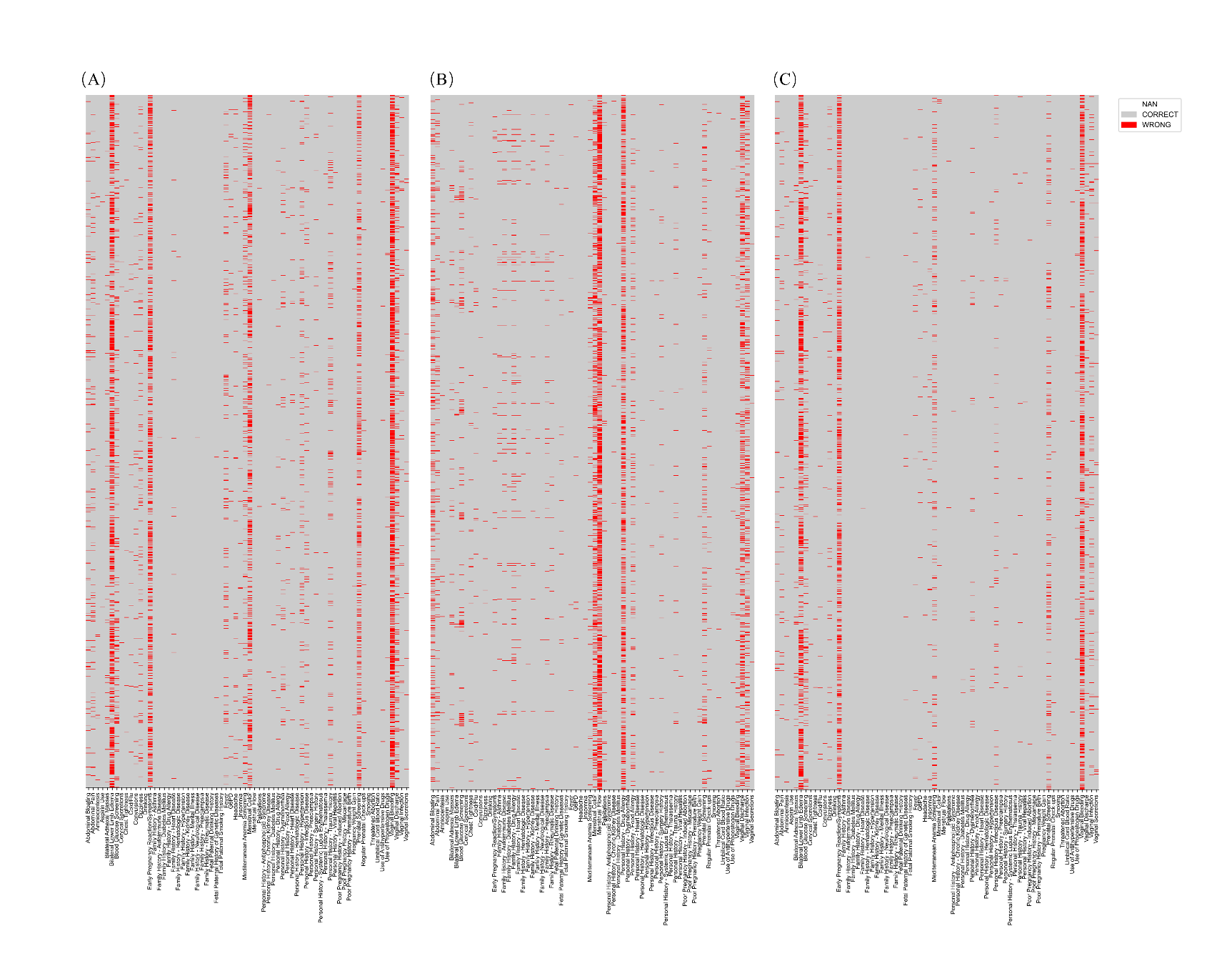


Figure S2 Overview of comparation between LLMs outputs and expert’s annotation when omitting corpus extraction step.


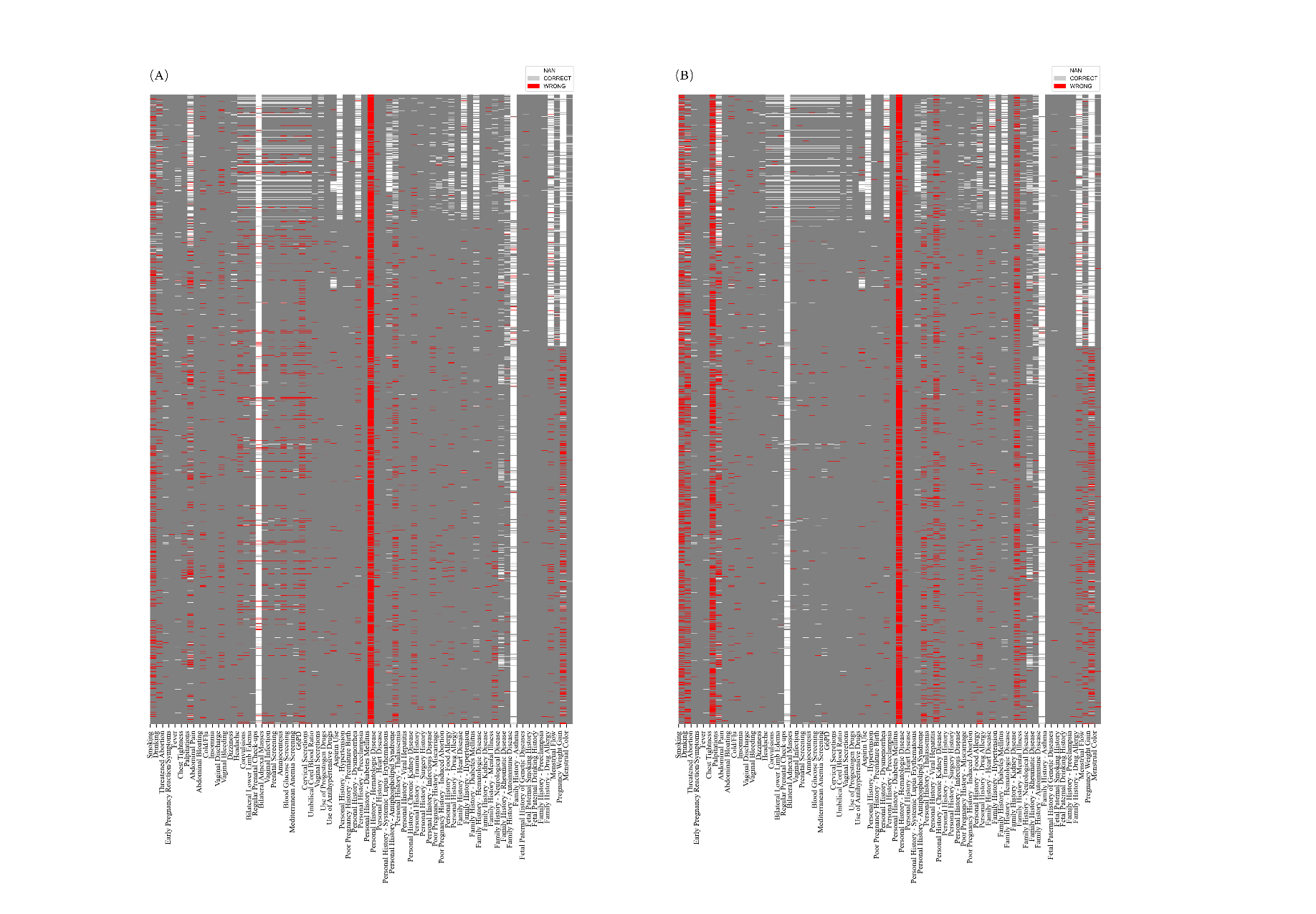

Supplement: Multimedia Appendix 2 [file jmir_v26i1e54580_app2.docx]
